# Cytohistopathological Study of Salivary Gland Lesions in Bundelkhand Region, Uttar Pradesh, India

**DOI:** 10.1155/2014/804265

**Published:** 2014-08-18

**Authors:** Anita Omhare, Sanjeev Kumar Singh, Jitendra Singh Nigam, Ankit Sharma

**Affiliations:** ^1^Department of Pathology, GSVM Medical College, Kanpur, Uttar pradesh 208012, India; ^2^Department of Pathology, M.L.B. Medical College, Jhansi, Uttar Pradesh 284128, India; ^3^Department of Pathology, Saraswathi Institute of Medical Sciences, Anwarpur, Pilkhuwa, Hapur, Uttar Pradesh 245304, India; ^4^Department of Pathology, D.D.U. Hospital, Harinagar, New Delhi 110066, India

## Abstract

*Background.* FNAC is a useful method for evaluating suspicious salivary glands lesions due to its low cost, minimum morbidity, rapid turnaround time, high specificity, and sensitivity.* Aim.* To know the frequency of the salivary gland lesions and cytohistological correlation in the Jhansi region, Uttar Pradesh, India.* Material and Methods.* In present study 124 cases were included and cytohistological correlation was made in 86 cases only. FNA was performed by using a 23/24-gauge needle without local anaesthesia. Air dried and 95% ethyl alcohol fixed wet smears were stained with Giemsa stain and Papanicolaou stain, respectively. Paraffin embedded tissue sections were stained with Haematoxylin and Eosin.* Results.* Parotid gland was the most commonly involved salivary gland. The commonest age group was 20 to 29 years, 30 to 39 years, and 60 to 69 years for nonneoplastic lesions, benign tumours, and malignant tumours, respectively. The overall male to female ratio was 1.17 : 1. The diagnostic accuracy of FNAC was 100%, 93.3%, and 88.2% for nonneoplastic lesions, benign tumours, and malignant tumours, respectively.* Conclusion.* The high accuracy, sensitivity, and specificity of FNAC confirm that preoperative cytology is a useful, quick, reliable diagnostic technique for rapid diagnosis and suitable for developing countries.

## 1. Introduction

Salivary glands are exocrine organs responsible for production and secretion of saliva and consist of the parotid, submandibular, sublingual, and the minor glands that are numerous and widely distributed throughout the mouth and oropharynx [1]. Salivary glands neoplasms account for 6% of all head and neck tumors [1]. Fine needle aspiration cytology (FNAC) is a useful method for evaluating suspicious salivary glands lesions due to its low cost, minimum morbidity, rapid turnaround time, high specificity, and sensitivity [2]. By cytological examination, lesions can be divided into inflammatory, reactive, benign, or malignant and, if possible, specific diagnosis is given which helps the clinicians in planning the management of the lesion [3]. The present study was taken to know the frequency of the salivary gland lesions especially in reference to malignancy in the Jhansi region, cytological evaluation of salivary gland masses by FNAC methods with regard to sensitivity, specificity and accuracy, and cytohistopathological correlation of salivary glands masses.

## 2. Material and Methods

Present study was carried out in the Department of Pathology, MLB Medical College, Jhansi, retrospectively and prospectively. Cases were selected from the patients attending ENT department or were admitted in the wards and also from the records presented with salivary gland swelling in the parotid, submandibular, and submental region. In the present study, 132 cases of salivary gland swelling are included in which cytological and histological studies were done. Eight cases were excluded due to scanty, inadequate aspirate on FNAC; thus only 124 cases were included in this study and cytohistological correlation was made in 86 cases only and in the remaining 38 cases either cytology or histology was available. All patients were clinically evaluated by detailed history, clinical examination, and haematological and radiological investigations. FNA was performed from different sites of the salivary gland swelling using a 10 mL disposable syringe and 23/24-gauge needle without local anaesthesia. FNA air-dried smears were stained with Giemsa stain and wet smears fixed in 95% ethyl alcohol were stained with Papanicolaou stain. Paraffin embedded tissue sections obtained from salivary gland tissue were stained with haematoxylin and eosin and few special stains were performed whenever required. Salivary gland lesions were studied under the three groups including nonneoplastic lesions and benign and malignant tumors.

## 3. Results

**Figure 1:**
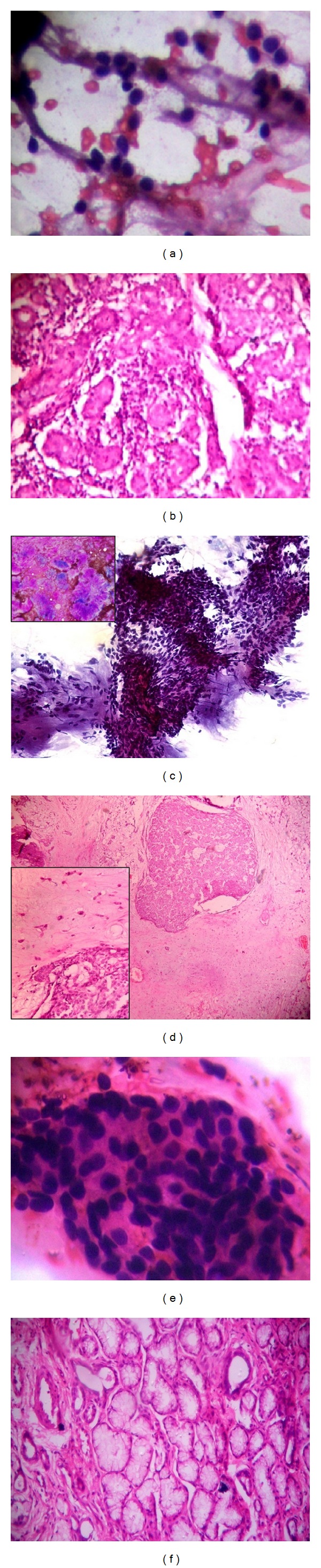
(a) Chronic sialadenitis: hypocellular smear with background lymphocytes and small cohesive group of ductal cells (Giemsa ×400). (b) Chronic sialadenitis: periductal fibrosis accompanied by chronic inflammatory cell infiltrate and acinar atrophy (H&E ×400). (c) Pleomorphic adenoma: the matrix is in slight magenta colour with myoepithelial cells present individually and in clusters (Giemsa ×400). (d) Pleomorphic adenoma: ductal structures are surrounded by abluminal myoepithelial cells and hyalinized, myxoid, and chondromyxoid stroma (H&E ×400). (e) Monomorphic adenoma: fragmented groups of haphazard arranged cells as squamous morules and intercellular matrix droplets (Giemsa ×400). (f) Monomorphic adenoma: aggregates of tumor cells were arranged as an inner layer of luminal epithelial cells and surrounded by an outer layer of myoepithelial cells (H&E ×400).

**Figure 2:**
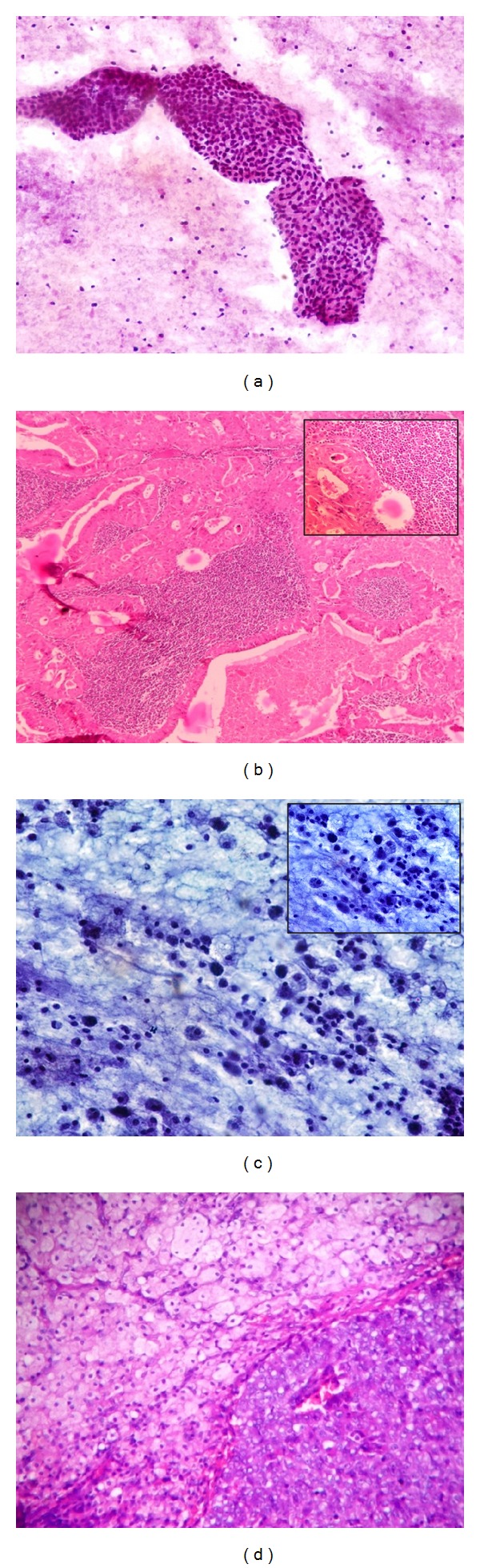
(a) Warthin tumor: the characteristic findings include cohesive flat sheets of oncocytes, lymphocytes, and a granular proteinaceous background (Giemsa ×400). (b) Warthin tumor: oncocytic epithelium with cuboidal basal cell to columnar luminal cell dense lymph node-like stroma (H&E ×400, Inset, H&E ×400). (c) Mucoepidermoid carcinoma: cells with abundant squamoid to vacuolated cytoplasm and large nuclei with a prominent nucleoli and necrotic background (Giemsa ×400). (d) Mucoepidermoid carcinoma: cluster of epidermoid cells with numerous clear cells (H&E ×400).

In the present study, nonneoplastic lesions accounted for 53.22% (66/124), followed by 31.45% (39/124) benign tumours and 15.32% (19/124) malignant tumours. Commonest gland involved was parotid (48.3%, 60/124), followed by submandibular gland (41.2%, 51/124) and minor salivary glands (10.4%, 13/124) whereas no case of sublingual salivary gland lesion was observed in the present study. Age range for nonneoplastic lesions was 8 years to 68 years with commonest age group being 20 to 29 years. Male : female ratio was 3.7 : 1. Age range for neoplastic lesions was 18 years to 78 years with commonest age group for benign neoplasms being 30 to 39 years, and, for malignant neoplasms, it was 60 to 69 years. Male : female ratio was 0.34 : 1. The overall male to female ratio was 1.17 : 1. In nonneoplastic lesions, 38 lesions involved the submandibular gland (57.5%, 38/66), 20 lesions involved the parotid gland (30.35%, 20/66), and 8 lesions involved the minor salivary gland (12.1%, 8/66). Chronic sialadenitis was the commonest lesion Figures 1(a) and 1(b) (63.64%, 42/66) followed by benign cysts (16.67%, 11/66), suppurative sialadenitis (7.57%, 5/66), and tubercular sialadenitis (6%, 4/66). In neoplastic lesions, most commonly involved site was the parotid gland (68.9%, 40/58) followed by submandibular gland (22.42%, 13/58) and minor salivary gland (8.62%, 5/58). In benign tumours, pleomorphic adenoma accounted for maximum number of cases (66.6%, 26/39), followed by monomorphic adenoma (25.6%, 10/39), haemangioma (5.1%, 2/39), and Warthin's tumour (2.5%, 1/39) Figures 1(c)–1(f), 2(a), and 2(b). In malignant lesions, mucoepidermoid Figures 2(c) and 2(d) carcinoma was the most common malignant tumour (42.1%, 8/19) followed by malignant mixed tumour (21%, 4/19), acinic cell carcinoma (21%, 4/19), adenoid cystic carcinoma (10.5%, 2/19), and adenocarcinoma (5.3%, 1/19). In the present study, both cytology and histology were carried out in 86 cases only and a correlation was done for sensitivity, specificity, and diagnostic accuracy. The diagnostic accuracy of FNAC for the nonneoplastic lesions, benign tumours, and malignant tumours was 100%, 93.3%, and 88.2%, respectively, and overall diagnostic accuracy was 95.3%. Positive predictive value and negative predictive value for neoplastic tumours were 88.2% and 97.1%, respectively. In nonneoplastic lesions, the specific diagnosis of all 39 cases by FNAC was correlated with histopathological findings. In benign tumours (30 cases) cytological diagnosis of 28 cases was consistent with histopathological diagnosis. Two cases reported as pleomorphic adenoma and Warthin's tumor on FNAC turn to malignant mixed tumour and low grade mucoepidermoid carcinoma, respectively, on histopathological examination. In malignant group, cytological diagnosis of 15 cases was consistent with histopathological diagnosis. In 2 cases cytological diagnosis of malignant mixed tumors turns to pleomorphic adenoma on histopathological examination.

## 4. Discussion

In the diagnosis of salivary gland lesions, FNAC has gained the popularity as diagnostic tool due to its low cost and safe procedure with minimal risk to the patient [4] and aid to the clinicians in the management planning. The rate of unsatisfactory samples on FNAC is varied from 3% to 12% [5–8]. In present study it was 6.4%. This difference may be due to inexperience of the pathologist and sampling errors. Nguansangiam et al. observed the age range from 6 to 100 years with mean age 53 years and female predominance [5]. As opposed to this, present study observed the age range of 8 to 68 years with mean age of 40 years with male predominance which was similar to the study done by Choudhury et al. [9]. The rate of nonneoplastic lesion in this study was 53.22%. It is in concordance with those of other studies, ranging from 20% to 72.9% [5, 10–12]. In the present study most common age group for nonneoplastic lesions was 20 to 40 years and male to female ratio was 3.7 : 1. Most common nonneoplastic lesion was chronic sialadenitis followed by benign cyst and most of the nonneoplastic lesions involved the submandibular gland and this similar finding is also observed by Atula et al. [13]. In the present study, benign neoplasms accounted for 39 cases (31.45%). The rate of benign neoplasm was lower than other reports which ranged from 49 to 83% [5–9]. We observed the pleomorphic adenoma as the commonest benign neoplasm followed by monomorphic adenoma and the predominance of these two benign neoplasms was similar to those previously reported number of studies [5, 10–12]. Various authors have reported that the incidence of malignant tumours ranged from 15% to 32% [10, 11], and in the present study it accounted for 15.32% whereas Nguansangiam et al. have found the lower rate of malignant neoplasms [5]. In our study, the most common malignant salivary gland tumor was mucoepidermoid carcinoma which accounted for 42.1% of all malignant neoplasms followed by malignant mixed tumours. As compared to this, Nguansangiam et al. have found that lymphoma is the commonest primary malignant salivary gland tumors followed by mucoepidermoid carcinoma [5]. Parotid gland was observed as the commonest site of salivary gland neoplasms; 68.9% (40/58) of all salivary gland neoplasms involved the parotid gland in this series. Almost similar distribution of salivary gland neoplasms in the parotid gland has also been described by Choudhury et al. [9]. The present study showed a false positive rate of 2.3%; it is in keeping with those of other studies, ranging from 0 to 4.7% [4, 14]. The false negative rate in the present study was also 2.3%, which was in concordance with other studies, reporting a range of 2.2% to 24.5% [5, 14, 15]. Diagnosis of low grade mucoepidermoid carcinoma is difficult because it may be misdiagnosed as chronic sialadenitis, Warthin's tumor, mucous retention cysts, and adenomatoid hyperplasia of the mucous salivary gland as observed in the present study [15]. In our study, sensitivity, specificity, positive predictive value (PPV), and negative predictive value (NPV) of preoperative FNA cytology of salivary gland tumours were 88.2%, 97.1%, 88.2%, and 97.1%, respectively; however, overall diagnostic accuracy for all salivary gland lesions was 95.3%, indicating good results compared with those previously reported in various studies [2, 6, 7, 11, 14].

## 5. Conclusion

The high accuracy, sensitivity, and specificity of FNAC confirm that preoperative cytology is a useful, quick, reliable diagnostic technique for rapid and early diagnosis and we also conclude that it is simple and cost-effective diagnostic tool suitable for developing countries.
